# Cutaneous Metastasis From Gallbladder Carcinoma: A Rare Case

**DOI:** 10.7759/cureus.73633

**Published:** 2024-11-13

**Authors:** Devansh A Patel, Dinesh G Prasad, Nandan J Patel, Saakshi P Kothari, Alay S Thoria

**Affiliations:** 1 General Surgery, Surat Municipal Institute of Medical Education and Research, Surat, IND; 2 General Surgery, Government Medical College, Surat, Surat, IND

**Keywords:** adenocarcinoma, cutaneous metastasis, gallbladder carcinoma, scrotal involvement, ulcerative lesion

## Abstract

The occurrence of cutaneous metastasis from internal malignancies is relatively rare. Furthermore, cutaneous metastasis from gallbladder carcinoma is an infrequent phenomenon, with only a few reported cases.

Here, we report a case of a 45-year-old male with cutaneous metastasis from primary gallbladder carcinoma, initially presenting solely with a scrotal lesion. The lesion was initially misdiagnosed. Later, an abdominal ultrasound performed for another purpose incidentally revealed a mass in the gallbladder, which was subsequently confirmed as gallbladder carcinoma by CT scan (computed tomography scan). Following the diagnosis of gallbladder carcinoma, an incisional biopsy of the lesion was performed due to the suspicion of cutaneous metastasis. Histopathological analyses and the immunohistochemical staining of the biopsy specimen confirmed it as metastatic adenocarcinoma.

As for the neoplasm of the gallbladder, skin is a comparatively infrequent site of metastasis, with only a few recorded incidents. Clinicians, especially in regions with a higher incidence of gallbladder carcinoma, should always consider cutaneous metastasis as a differential while treating any abnormal skin lesion in patients with gallbladder carcinoma.

## Introduction

Carcinoma of the gallbladder, accounting for more than 50% of all biliary tract carcinomas, is an aggressive tumor with a very low five-year survival rate and poor prognosis, owing to its late presentation, advanced stage at diagnosis, and unfavorable anatomical position [1]. Although rare globally, gallbladder carcinoma shows higher prevalence in certain regions, including South American countries like Chile, Bolivia, and Ecuador and some Asian countries like India, Pakistan, Japan, and South Korea [2].

Gallbladder carcinoma can spread by direct extension, as well as through lymphatic, hematogenous, intraductal, and neural routes. The liver is the most involved structure by direct extension, followed by lymph nodes. Extra-abdominal metastasis is rare, most commonly involving the lungs. Metastasis to the brain [3], orbit [4], breast [5], skin, and bones is also known to occur. Cutaneous metastasis of primary gallbladder cancer has an incidence of 0.7-0.9% [6], with only a few cases documented. Reported sites of cutaneous metastases from gallbladder carcinoma include neck, chest, abdomen, thigh, and scalp [5,6]. The mechanism that allows the development of cutaneous metastasis from gallbladder carcinoma is poorly understood, although pathways such as lymphatic and hematogenous spread have been suggested.

The occurrence of cutaneous metastasis usually indicates widespread disease with a poor prognosis. Patients of gallbladder carcinoma tend to remain asymptomatic in the early stages, and by the time symptoms appear, the tumor often becomes advanced and unresectable. In such advanced cases, curative treatment options remain limited. The focus of management remains on palliative care, optimizing quality of life, and managing symptoms effectively. Multidisciplinary care plays a vital role in determining the management of such patients. As of now, there are no specific guidelines focused solely on the management of gallbladder carcinoma with cutaneous metastasis. First-line therapy for metastatic gallbladder carcinoma includes chemotherapy aimed at controlling tumor spread and improving quality of life. Surgical intervention is not generally considered in metastatic gallbladder carcinoma. However, excision of the cutaneous lesion may be considered for palliative reasons in case the lesion causes significant discomfort or for cosmetic reasons.

In this case report, we present a rare case of gallbladder carcinoma with cutaneous metastasis, whose initial presentation was an unusual cutaneous lesion over the scrotum. The patient was treated with chemotherapy as the tumor had already metastasized.

## Case presentation

A 45-year-old male presented with a lesion on the right side of the scrotum that had persisted for over a month and was accompanied by pain and tingling sensation. The lesion progressively increased in size over the month, and the pain was burning in nature. The patient was otherwise asymptomatic and reported no complaints of weight loss, anorexia, and abdominal pain.

Personal history suggested that the patient had a 20-year history of alcohol consumption and has maintained abstinence for the past five to six years. The patient has also been consuming tobacco over the last 15 years.

**Figure 1:**
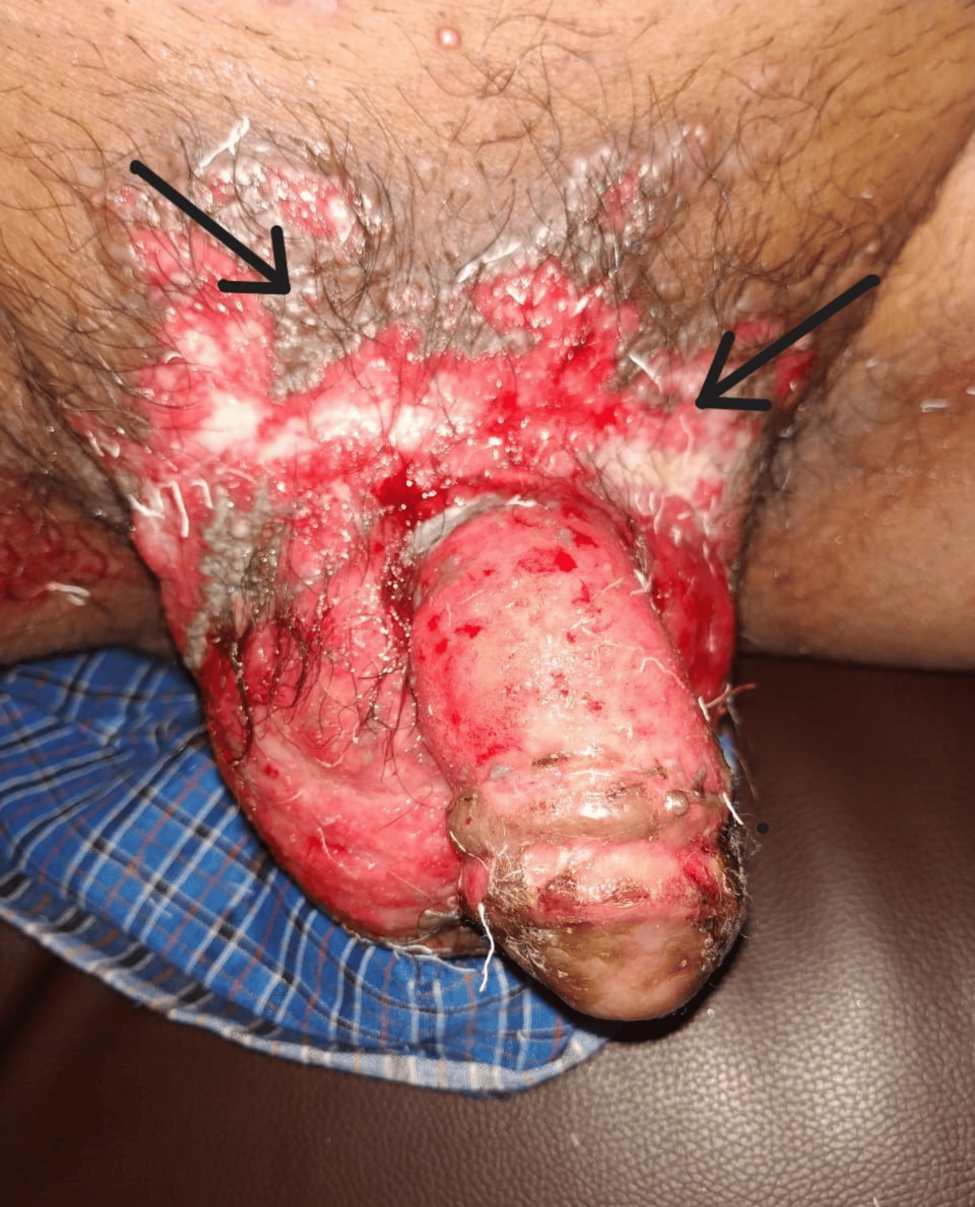
Ulcerative cutaneous lesion involving scrotum, pubic region, and penile skin (black arrow).

On initial physical examination, an irregular, well-circumscribed, ulcerated swelling of approximately 3 cm x 2 cm in size with an erythematous base was seen over the right side of the scrotum near the base of the penis. The swelling was indurated, non-mobile, tender on touch, associated with oozing, and not adhered to underlying tissue. The size of the ulcerative lesion gradually increased to approximately 8 cm x 3 cm, causing involvement of the pubic region and the penile skin (Figure 1).

In addition to the lesion over the scrotum, the right-sided testis appeared to be smaller in size during palpation. Other physical findings were found to be normal, including the absence of icterus and any palpable abdominal mass or lymph nodes. No abnormal findings were present on systemic examination involving the respiratory and cardiovascular systems.

**Figure 2:**
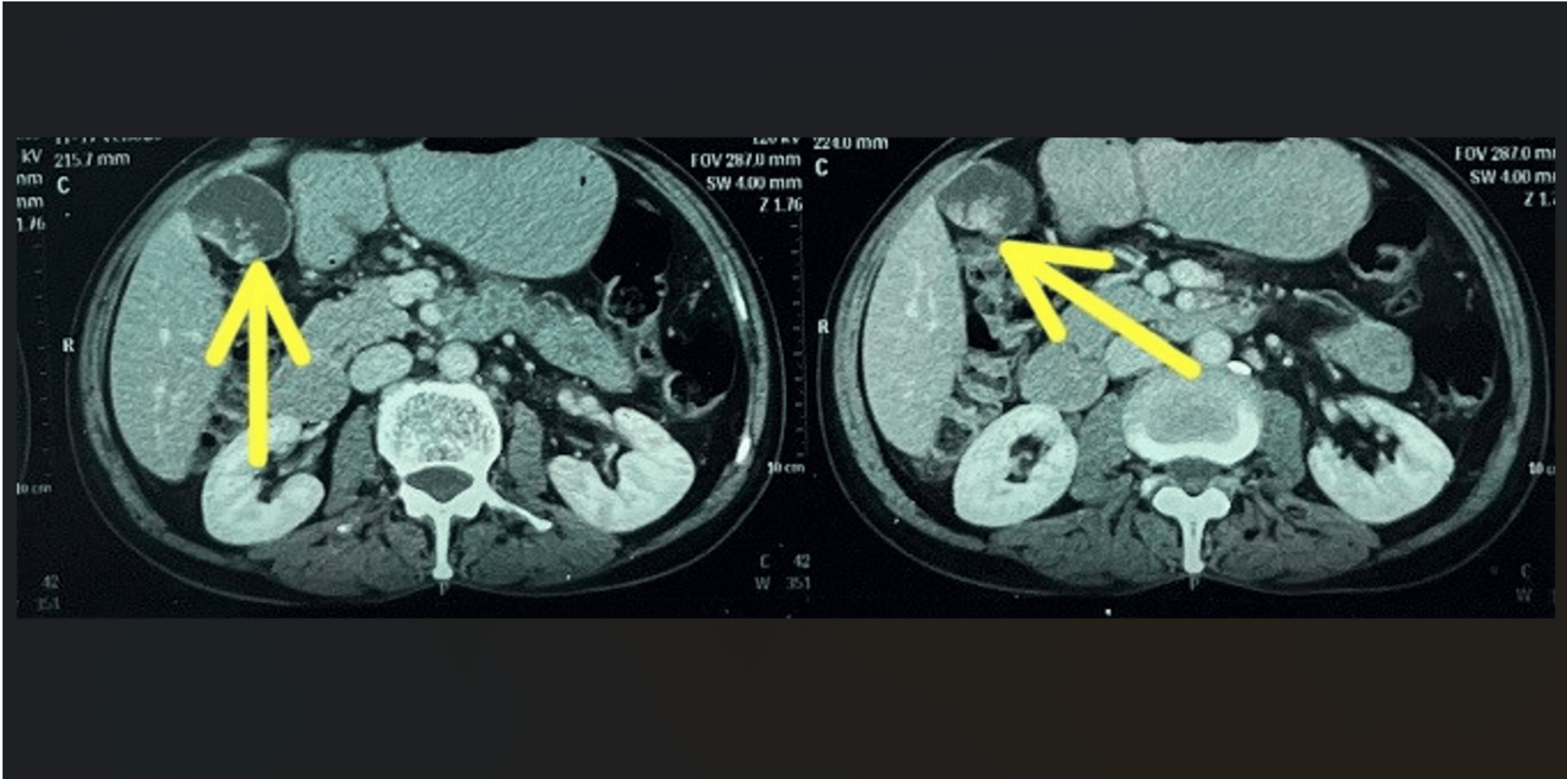
CT scan demonstrating a mass in the gallbladder during the portal venous phase (yellow arrow). CT: computed tomography

Initially, an ultrasound of the inguinoscrotal region was performed, and the findings were suggestive of bilateral epididymitis with associated funiculitis along with atrophy of the right testis. Additionally, an abdominal ultrasound was conducted to investigate potential causes of epididymitis, such as tuberculosis, which led to the incidental finding of a hyperechoic lesion involving the body and fundus region of the gallbladder. A computed tomography (CT) scan was later performed to confirm the gallbladder pathology. It revealed an irregular, polypoidal, heterogeneously enhanced soft tissue lesion involving the dependent wall of the body and fundus of the gallbladder, suggesting a primary malignant etiology, along with the involvement of lymph nodes (Figure 2).

Laboratory analysis showed no significant rise in aspartate aminotransferase (AST), alanine aminotransferase (ALT), alkaline phosphatase (ALP), and serum bilirubin levels. Other liver function tests were also within normal limits. Serological tests were negative for hepatitis-B surface antigen (HBsAg) and hepatitis-C virus antibodies.

The scrotal lesion was initially misdiagnosed and treated as a bacterial infection with antibiotics. When the antibiotics showed no significant response, acyclovir was started, suspecting the lesion might be due to a herpes infection. However, the treatment showed no improvement. Following the incidental diagnosis of gallbladder carcinoma, suspicions of cutaneous metastasis were raised, and an incisional biopsy was performed to rule out this possibility.

**Figure 3:**
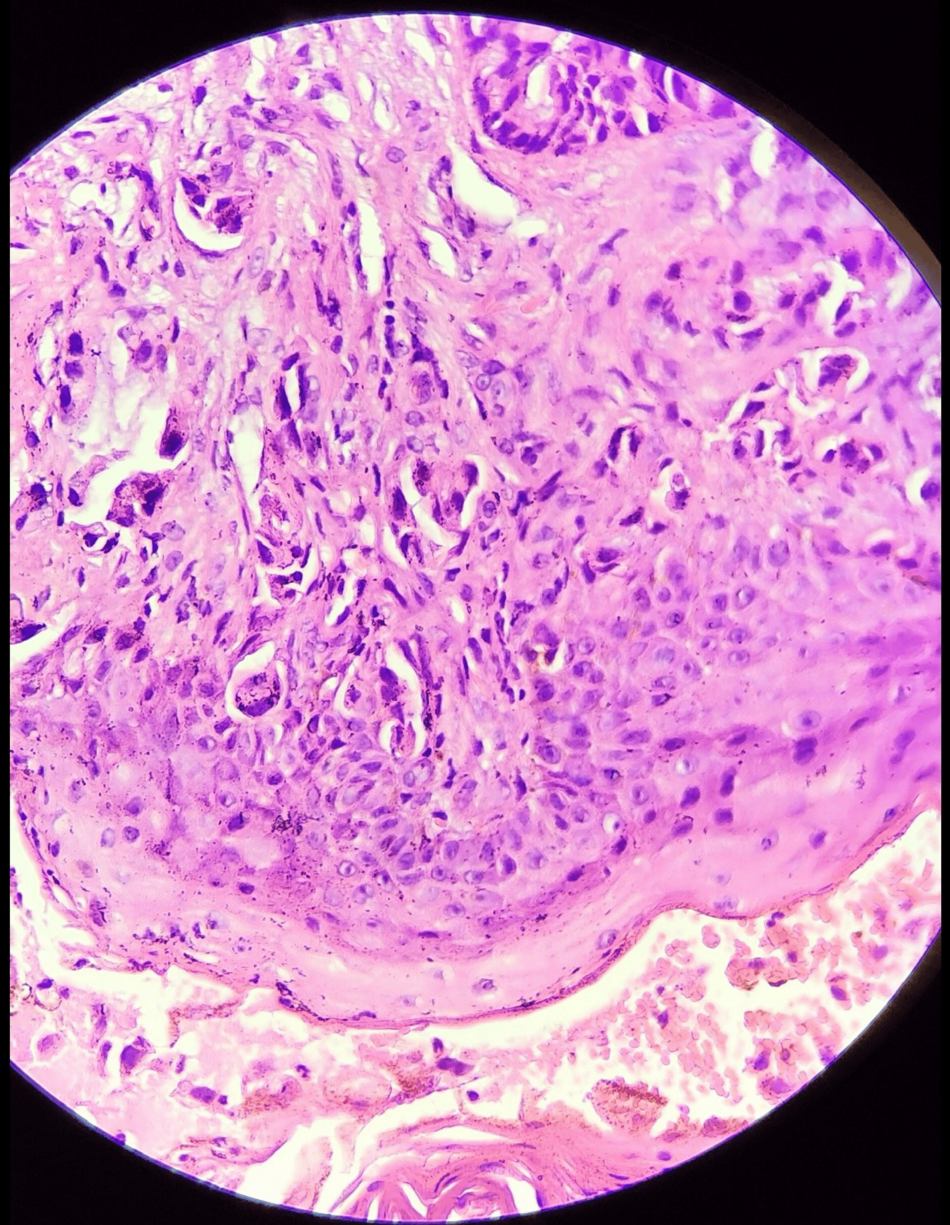
Histopathological findings reveal malignant cells arranged in a glandular pattern in the subepithelial tissue, along with inflammatory infiltrates of polymorphs and lymphocytes, as well as areas of hemorrhage and congestion, suggestive of adenocarcinoma. Staining was performed using H&E at 400x magnification. H&E: Hematoxylin and eosin

Histopathological examination showed infiltration of the sub-epithelial layer with malignant cells arranged in a glandular pattern with individual tumor cells having a high nuclear-to-cytoplasmic (N:C) ratio, irregular nuclear membrane, prominent nucleoli with a scanty to moderate amount of cytoplasm, along with inflammatory infiltrates of polymorphs and lymphocytes (Figure 3).

**Figure 4:**
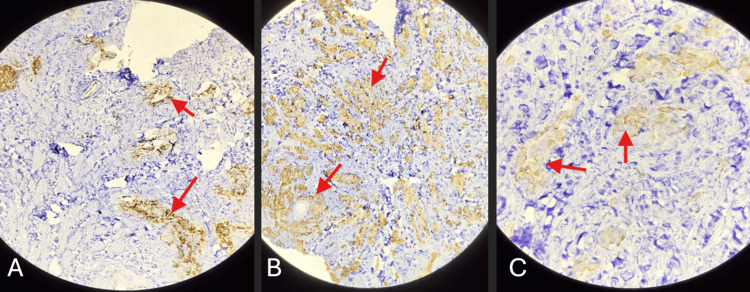
Immunohistochemical (IHC) staining of biopsy sample of cutaneous lesion. (A) Immunohistochemical staining shows positive CEA expression; (B) Immunohistochemical staining shows positive CK-7 expression; (C) Immunohistochemical staining shows positive CK-20 expression. CEA: carcinoembryonic antigen; CK: cytokeratin

Immunohistochemical (IHC) staining of the tumor cells in skin biopsy was positive for carcinoembryonic antigen (CEA), cytokeratin 7 (CK7), and cytokeratin 20 (CK20) (Figure 4A, 4B, 4C). This helped in forming the diagnosis of metastatic adenocarcinoma. The patient received gemcitabine and cisplatin therapy, as determined by the surgeon and oncologist. At the time of the last follow-up, the patient was stable and receiving palliative care.

## Discussion

Gallbladder carcinoma is the most common biliary tract cancer, making up more than 50% of all biliary tract cancers, and is the sixth most common gastrointestinal malignancy [7]. Gallbladder carcinoma is a chronic inflammatory disease promoted by different risk factors, including gallstone disease, sedentary lifestyle, smoking, alcohol consumption, metabolic disorders, high-fat diet, hypercholesterolemia, and some types of infection [8]. Our patient had a notable history of alcohol intake spanning approximately 20 years. The five-year survival rate of patients with metastatic gallbladder carcinoma is less than 5%. Moreover, patients of gallbladder cancer with cutaneous metastases have a median survival period of 7.5 months [9]. Gallbladder carcinoma is usually asymptomatic in its early stages, but some patients develop symptoms such as pain in the right hypochondriac region, jaundice, pruritus, weight loss, anorexia, dark urine, and pale stool. Symptoms are usually non-specific and can be mistaken for gallstone disease or cholecystitis. Our patient was asymptomatic other than the cutaneous lesion at the time of the presentation.

Though the exact mechanism remains unclear, the homing mechanism may provide some clues to understanding the metastatic process. The interaction between tumor cells and certain factors secreted from the dermis and epidermis might be involved in the skin-homing mechanism of metastatic cells [9]. Understanding the molecular mechanisms underlying the metastatic process could provide some valuable insights into the metastatic behavior of gallbladder carcinoma and can also provide potential therapeutic targets to prevent metastasis.

The most common histological type of gallbladder cancer with cutaneous metastasis is adenocarcinoma [10]. Metastatic skin lesions are usually non-tender, erythematous, nodular, and either cutaneous or subcutaneous [6]. Sometimes, metastatic lesions may even resemble cellulitis, herpes zoster, condyloma, epidermal inclusion cyst, and ulcerative lesions [6]. The sharing of similarities with other skin lesions increases the chances of misdiagnosis as well as late diagnosis and false treatment. Rarely, cutaneous lesions may be the initial presentation of gallbladder malignancy. Any abnormal cutaneous lesion in patients with gallbladder carcinoma should be ruled out for metastasis. Cutaneous metastases from the gallbladder cancer indicate an advanced stage of tumor along with a poor prognosis.

Histopathological examination is necessary for the diagnosis of cutaneous metastases. Fine needle aspiration cytology (FNAC) could be an alternative, but biopsy is the method of choice to ensure a successful histopathological examination [9]. Immunohistochemical staining plays a critical role in detecting the origin of metastases, especially when the initial presentation is atypical. For gallbladder carcinoma, IHC markers such as CK7, CK20, and CEA are particularly useful. IHC markers should always be correlated with radiological and clinical findings. CT scan, MRI, and PET scan can help identify the site and extent of the primary lesion as well as help in staging the disease. 

Surgery is the only likely curative option, but it can be considered in only 10-30% of patients because of the local and distant spread of the tumor and advanced stage at presentation [11]. Patients of gallbladder carcinoma with distant metastasis carry poor prognosis, and management of such cases is typically palliative. As per the National Comprehensive Cancer Network (NCCN) guidelines for gallbladder cancer, chemotherapy using durvalumab with gemcitabine and cisplatin is the first line of treatment in unresectable cases [12]. Only gemcitabine with cisplatin or gemcitabine with paclitaxel can also be used. If an unresectable tumor progresses during or after the initial therapy, then fluoropyrimidine-based regimens like FOLFOX (5-fluorouracil, leucovorin, and oxaliplatin) or FOLFIX (5-fluorouracil, leucovorin, and irinotecan) may be tried. Targeted therapies may also be tried in patients with specific mutations, such as pemigatinib for FGFR2 fusions and ivosidenib for IDH1 mutation. Immunotherapy using nivolumab or durvalumab may also be tried. Radiotherapy or surgical excision of cutaneous lesions may also be considered for palliation if causing discomfort or cosmetic blemish. Practicing multidisciplinary care in such patients can help form a treatment plan that best suits the patient.

## Conclusions

In conclusion, this case report illustrates the infrequent occurrence of cutaneous metastasis in gallbladder carcinoma, highlighting its potential to complicate diagnosis and management. The scrotum as a site for cutaneous metastasis from gallbladder carcinoma is an uncommon occurrence. Documentation of unusual metastatic sites is essential for enhancing clinical awareness and knowledge within the medical community. This case further highlights the critical importance of immunohistochemical markers such as CK7, CK20, and CEA for establishing the diagnosis of cutaneous metastasis in patients of gallbladder carcinoma. Such unusual presentations should prompt physicians to maintain a broad differential diagnosis, including cutaneous metastasis, as early recognition is essential for timely intervention in these cases. 
